# Inhibition of Mcl-1 Synergistically Enhances the Antileukemic Activity of Gilteritinib and MRX-2843 in Preclinical Models of *FLT3*-Mutated Acute Myeloid Leukemia

**DOI:** 10.3390/cells11172752

**Published:** 2022-09-03

**Authors:** Shuangshuang Wu, Holly Edwards, Deying Wang, Shuang Liu, Xinan Qiao, Jenna Carter, Yue Wang, Jeffrey W. Taub, Guan Wang, Yubin Ge

**Affiliations:** 1 Department of Pediatric Hematology, The First Hospital of Jilin University, Changchun 130021, China; 2 National Engineering Laboratory for AIDS Vaccine, School of Life Sciences, Jilin University, Changchun 130012, China; 3 Department of Oncology, Wayne State University School of Medicine, Detroit, MI 48202, USA; 4 Molecular Therapeutics Program, Karmanos Cancer Institute, Wayne State University School of Medicine, Detroit, MI 48201, USA; 5 The Tumor Center of the First Hospital of Jilin University, Changchun 130021, China; 6 Cancer Biology Graduate Program, Wayne State University School of Medicine, Detroit, MI 48201, USA; 7 MD/PhD Program, Wayne State University School of Medicine, Detroit, MI 48201, USA; 8 Department of Pediatrics, Wayne State University School of Medicine, Detroit, MI 48202, USA; 9 Division of Pediatric Hematology and Oncology, Department of Pediatrics, Children’s Hospital of Michigan, Detroit, MI 48201, USA; 10 Department of Pediatrics, Central Michigan University College of Medicine, Mt. Pleasant, MI 48859, USA

**Keywords:** acute myeloid leukemia, Mcl-1, AZD5991, FLT3, FLT3-ITD, MRX-2843, gilteritinib

## Abstract

*FMS-like tyrosine kinase 3* (*FLT3*)-internal tandem duplication (*FLT3*-ITD) mutations occur in about 25% of all acute myeloid leukemia (AML) patients and confer a poor prognosis. FLT3 inhibitors have been developed to treat patients with *FLT3*-mutated AML and have shown promise, though the acquisition of resistance occurs, highlighting the need for combination therapies to prolong the response to FLT3 inhibitors. In this study, we investigated the selective Mcl-1 inhibitor AZD5991 in combination with the FLT3 inhibitors gilteritinib and MRX-2843. The combinations synergistically induce apoptosis in AML cell lines and primary patient samples. The FLT3 inhibitors downregulate *c-Myc* transcripts through the suppression of the MEK/ERK and JAK2/STAT5 pathways, resulting in the decrease in c-Myc protein. This suppression of c-Myc plays an important role in the antileukemic activity of AZD5991. Interestingly, the suppression of c-Myc enhances AZD5991-inudced cytochrome *c* release and the subsequent induction of apoptosis. AZD5991 enhances the antileukemic activity of the FLT3 inhibitors gilteritinib and MRX-2843 against *FLT3*-mutated AML in vitro, warranting further development.

## 1. Introduction

FMS-like tyrosine kinase 3 (FLT3) is expressed on the cell surface of hematopoietic progenitor cells and on most AML cells from patients [1]. *FLT3*-internal tandem duplication (*FLT3*-ITD) mutations occur in about 25% of all AML patients [2]. Another 6–8% of AML patients have mutations in the tyrosine kinase domain (*FLT3*-TKD) [3], though the prognosis is similar to those with *FLT3*-wild type (*FLT3*-wt) AML [4,5,6]. *FLT3*-ITD results in the constitutive activation of the downstream MEK/ERK, PI3K/AKT, and JAK2/STAT5 pathways, which promote the survival and proliferation of AML cells [7]. Therefore, FLT3 inhibitors hold great promise for treating *FLT3*-mutated AML.

Gilteritinib (ASP-2215) is a dual inhibitor of FLT3 and AXL that is approved by the US FDA for treating adult AML patients with relapsed/refractory AML and a *FLT3* mutation. MRX-2843 (also known as UNC2371) is a novel small molecule that inhibits both MERTK and FLT3 and their downstream signaling pathways [8]. MERTK is overexpressed in the majority of AML patient samples compared to normal bone marrow (>80%) [9]; thus, it can target AMLs with and without *FLT3*-ITD. In vitro studies have shown that targeting MERTK has therapeutic potential [9]. Preclinical studies show that MRX-2843 has potent antileukemic activity against AML cells expressing MERTK and/or *FLT3*-ITD both in vitro and in vivo [8]. Furthermore, MRX-2843 is currently being investigated in Phase I/II clinical trials for relapsed/refractory AML (NCT04872478 and NCT04946890). 

FLT3 inhibition shows a good initial response, though one that is not sustained [10]. Thus, combination therapies may help prolong a sustained response to FLT3 inhibitors. FLT3 inhibitors have been shown to downregulate Mcl-1 [11,12], which plays a critical role in leukemic cell survival [13]. We previously reported that gilteritinib treatment substantially decreased Mcl-1 protein levels in *FLT3*-ITD AML cells [14], which could sensitize the cells to Mcl-1 inhibition [15]. Additionally, we found that gilteritinib decreases c-Myc protein levels [14], and c-Myc and Mcl-1 have been shown to cooperatively support cancer cell survival [16,17,18,19]. Furthermore, we recently reported that c-Myc plays an important role in the antileukemic activity of the Mcl-1 inhibitor AZD5991 [15]. Therefore, we hypothesized that Mcl-1 inhibition would synergize with FLT3 inhibitors against *FLT3*-mutated AML cells. In this study, we investigated the antileukemic activity of the selective Mcl-1 inhibitor AZD5991 in combination with the FLT3 inhibitors gilteritinib and MRX-2843, as well as the role c-Myc plays in the antileukemic interactions between AZD5991 and FLT3 inhibitors. 

## 2. Materials and Methods

### 2.1. Drugs

Gilteritinib, SCH772984, and AZD1480 were purchased from Selleck Chemicals (Houston, TX, USA). MRX-2843 and AZD5991 were purchased from MedChemExpress (Monmouth Junction, NJ, USA). 10058-F4 and Cytarabine (AraC) were purchased from AbMole Bioscience (Houston, TX, USA). 

### 2.2. Cell Culture

MOLM-13 was purchased from AddexBio (San Diego, CA, USA; 2012). MV4-11, THP-1, and HL-60 were purchased from the American Type Culture Collection, (ATCC, Manassas, VA, USA; 2006, 2014, and 2004, respectively). OCI-AML3 was purchased from the German Collection of Microorganisms and Cell Cultures (Braunschweig, Germany; 2011). All cell lines were cultured as previously described [20]. All cell lines were tested monthly for mycoplasma utilizing the PCR method described by Uphoff and Drexler [21] and were authenticated in 2017 at the Karmanos Cancer Institute’s Genomics Core via the PowerPlex^®^ 16 System (Promega, Madison, WI, USA). MV4-11 and MOLM-13 cells were treated with stepwise increasing concentrations of AraC to generate cells with acquired AraC resistance (designated MV4-11/AraC-R and MOLM-13/AraC-R), as previously described [14,15]. 

### 2.3. Clinical Samples

Primary AML patient samples and human umbilical cord blood samples were obtained from the First Hospital of Jilin University. Normal peripheral blood mononuclear cells (PBMCs) were donated by healthy individuals. Written informed consent was obtained in all cases as per the Declaration of Helsinki. Both the study and patient sample collections were approved by the Human Ethics Committee of The First Hospital of Jilin University (Ethical code # 2019-128). All AML patient samples were screened for gene mutations via PCR amplification and automated DNA sequencing, namely, *FLT3*-ITD, *NPM1*, *C-kit*, *CEBPA*, *IDH1*, *IDH2*, *SF3B1*, *TP53*, *ZRSR2*, *GATA2*, *KMT2A*, *SH2B3*, *TCF*, *TET2*, *RUNX1*, and *DNMT3A*. Cytogenetics and detection of fusion genes via real-time PCR were performed, as previously described [22,23]. Characteristics of the individual AML patients are listed in Table S1. Primary patient samples were purified with Ficoll-Hypaque density centrifugation and cultured as previously described [22,24].

### 2.4. Annexin V/Propidium Iodide (PI) Staining and Flow Cytometry Analyses

Cells were treated with the indicated drug(s) for up to 24 h. Annexin V-fluorescein isothiocyanate (FITC)/propidium iodide (PI) staining and flow cytometry analysis were performed as previously described [25]. Results are displayed as the percentage of annexin V positive cells, with all cell line experiments repeated in triplicate independently; displayed data are from one representative experiment. Experiments using primary patient samples were performed once in triplicate, due to limitations in sample availability. Combination index (CI) using CompuSyn software (Combosyn Inc., Paramus, NJ, USA) was calculated. CI < 1, CI = 1, and CI > 1 indicate synergistic, additive, and antagonistic effects, respectively [25,26].

### 2.5. Western Blots

Western blots were performed as previously described [27,28] using anti-Bax (50599-2-Ig), -ERK (16443-1-AP), -PARP (13371-1-AP), -β-actin (66009-1-Ig) (Proteintech, Rosemont, IL, USA), -p-AKT (T308; 13038S), -p-AKT (S473; 3787S), -cf-Caspase 3 (9661S), -p-STAT5 (Y694; 9359S), -AXL (8661s) (Cell Signaling Technologies, Danvers, MA, USA), -FLT3 (ab245116) (Abcam, Cambridge, MA, USA), -c-MYC (A5011), -Bak (A5068), -p-ERK (T202/Y204; A5036), -AKT (A5031), -cytochrome c (A5184), -VDAC1 (A5224), and -MERTK (A5615) (Bimake.cn, Shanghai, China) antibodies. The Odyssey Infrared Imaging System (Li-Cor, Lincoln, NE, USA) was used to visualize immunoreactive proteins, as described by the manufacturer. Western blots were repeated, at a minimum of three times, and one representative blot is displayed. The Odyssey V3.0 program (Li-Cor) was used to perform densitometry measurements.

### 2.6. Mitochondrial Fractionation/Cytochrome c Release

Mitochondria were isolated using the Mitochondria Extraction Kit (Solarbio Science and Technology, Beijing, China), as previously described [29]. 

### 2.7. shRNA Knockdown of Bak and Bax

The pMD-VSV-G and delta 8.2 plasmids were gifts from Dr. Dong at Tulane University. Bak, Bax, and non-target negative control (NTC) shRNA lentiviral constructs were purchased from Sigma Aldrich. Lentivirus production and transduction were carried out as previously described [30]. The Bak/Bax dual knockdown (Bak/Bax KD) and NTC MV4-11 cells were generated via combined Bak- and Bax- and NTC-shRNA lentiviral vectors, respectively, as previously reported [12,14,31].

### 2.8. CRISPR Knockdown

The lentiCRISPRv2 plasmid was a gift from Feng Zhang at the Broad Institute of MIT and Harvard (Addgene plasmid 52961). Guide RNAs were designed using the CRISPR design tool (http://crispr.mit.edu, accessed on 5 May 2022). The non-target control (NTC; 5′-GCACTACCAGAGCTAACTCA-3′) and c-Myc (5′-GTATTTCTACTGCGACGAGG-3′) vectors were generated using Feng Zhang’s protocol, which is available on Addgene’s website (www.addgene.org). Lentivirus production and transduction were carried out as described above in the “shRNA knockdown of Bak and Bax,” except psPAX2 (a gift from Didier Trono at the Swiss Institute of Technology, Addgene plasmid #12260) was used instead of delta 8.2.

### 2.9. Colony-Forming Assay

Colony-forming assays were carried out as previously described [32,33,34]. Cells were treated with gilteritinib or MRX-2843 and AZD-5991, alone or in combination, for 24 h, and then washed three times with PBS, plated in MethoCult (catalog number 04434; Stem Cell Technologies), and incubated for 10–14 days, according to the manufacturer’s instructions. Colony-forming units (CFUs) were visualized utilizing an inverted microscope. Colonies containing over 50 cells were counted. 

### 2.10. Real-Time RT-PCR

The cDNAs were prepared from 2 μg of total RNA (extracted using TRIzol (Thermo Fisher Scientific, Waltham, MA, USA)) using EasyScript All-in-One First-Strand cDNA Synthesis Super Mix (catalog number AE341; TransGen Biotech, Beijing, China). *c-Myc* transcripts were quantified using forward (5′- GTGGTCTTCCCCTACCCTCT-3′) and reverse (5′-CGAGGAGAGCAGAGAATCCG-3′) primers. *36B4* transcripts were quantified with forward (5′-CGACCTGGAAGTCCAACTAC-3′) and reverse (5′-ATCTGCTGCATCTGCTTG-3′) primers. Quantification was conducted using SYBR green and a LightCycler 480 real-time PCR machine (Roche Diagnostics), as per manufacturer’s instructions. Results of real-time PCR are presented as the mean of three independent experiments, normalized to *36B4* transcripts. Fold changes were calculated using the comparative Ct method [35].

### 2.11. Statistical Analysis

Unpaired *t*-test was used to compare differences between two groups, and statistical analyses were performed utilizing GraphPad Prism 9.0. Error bars represent standard error of the mean (s.e.m.); significance was set at *p* < 0.05. 

## 3. Results

### 3.1. AZD5991 Synergistically Enhances Apoptosis Induced by FLT3 Inhibition in FLT3-Mutated AML Cells

**Figure 1:**
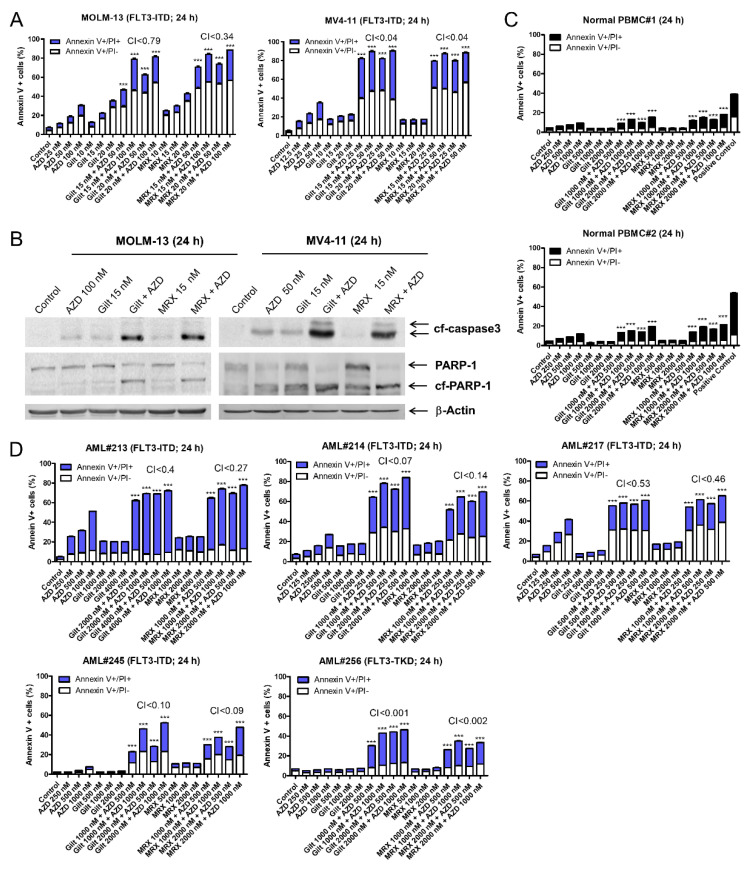
AZD5991 synergistically enhances apoptosis induced by gilteritinib and MRX-2843 in *FLT3*-mutated AML cells. (**A**) *FLT3*-ITD-positive MOLM-13 and MV4-11 cells treated with varying concentrations of AZD5991 (AZD), gilteritinib (gilt), or MRX-2843 (MRX), alone or in combination for 24 h were stained with annexin V-FITC/propidium iodide (PI) and analyzed using a flow cytometer. Combination Index (CI) values were calculated using CompuSyn software. CI < 1, CI = 1, and CI > 1 indicate synergistic, additive, and antagonistic effects, respectively. *** indicates *p* < 0.001. (**B**) MOLM-13 and MV4-11 cells were treated with AZD, gilt, AZD + gilt, MRX, or MRX + AZD for 24 h. Whole cell lysates were subjected to western blot analysis. (**C**) Normal peripheral blood mononuclear cells (PBMCs) treated with varying concentrations of AZD, gilt, AZD + gilt, MRX, or MRX + AZD for 24 h were stained with annexin V-FITC/PI and analyzed using a flow cytometer. *** indicates *p* < 0.001. (**D**) Primary AML patient sample cells were treated with varying concentrations of AZD, gilt, or MRX, alone or in combination, for 24 h; then, the cells were stained with annexin V-FITC/propidium iodide (PI) and analyzed using a flow cytometer. CI values were calculated using CompuSyn software. *** indicates *p* < 0.001.

To determine whether AZD5991 synergizes with FLT3 inhibition, *FLT3*-ITD positive AML cell lines MOLM-13 and MV4-11 were treated with the vehicle control, AZD5991, gilteritinib, or MRX-2843, alone or in combination, for 24 h. AZD5991 and gilteritinib treatment, as well as the AZD5991 and MRX-2843 treatment, resulted in significantly higher levels of cell death compared to the individual drug treatments and the vehicle control, as determined by the annexin V-FITC/PI staining and flow cytometry analyses. The CI values (CI < 0.79 and 0.04 for MOLM-13 and MV4-11, respectively) demonstrate that AZD5991 and gilteritinib or MRX-2843 synergize in inducing cell death in the *FLT3*-ITD AML cell lines (Figure 1A). The significant increase in annexin V positivity was accompanied by a substantial increase in the cleavage of caspase 3 and PARP (Figure 1B), demonstrating the induction of apoptosis. In contrast, the normal PBMCs treated with AZD5991 and gilteritinib or MRX-2843 at much higher concentrations (>10-fold) showed less than 20% annexin V positivity (Figure 1C). In addition, AZD5991 synergized with gilteritinib and MRX-2843 in the *FLT3*-mutated primary AML patient samples (Figure 1D). 

The AML cell lines, both *FLT3*-ITD and *FLT3*-wt, show variable protein levels of FLT3, AXL, and MERTK (Figure S1A). The treatment of *FLT3*-wt AML cell lines with AZD5991 in combination with gilteritinib or MRX-2843 also resulted in a synergistic induction of annexin V positivity (Figure S1B). This was also accompanied by caspase 3 and PARP cleavage (Figure S1C). Further, AZD5991 in combination with gilteritinib or MRX-2843 resulted in a synergistic induction of apoptosis in *FLT3*-wt primary AML patient samples (Figure S2). These results demonstrate that AZD5991 synergistically enhances the apoptosis induced by gilteritinib and MRX-2843 in *FLT3*-mutated AML cells, as well as *FLT3*-wt AML cells.

### 3.2. AZD5991 Alone and in Combination with Gilteritinib and MRX-2843 Significantly Reduces Colony-Forming Capacity of FLT3-Mutated AML Progenitor Cells

**Figure 2:**
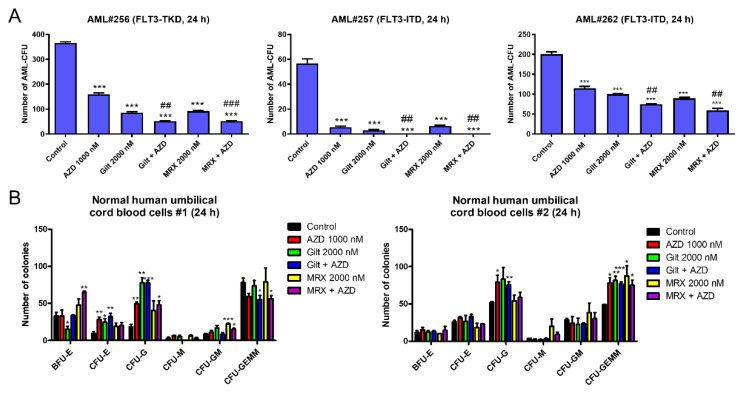
AZD5991 alone and in combination with gilteritinib or MRX-2843 significantly reduces colony-forming capacity of *FLT3*-mutated AML progenitor cells. (**A**,**B**) Primary AML patient samples (panel (**A**)) and normal human umbilical cord blood cells (panel (**B**)) were treated with AZD5991 (AZD), gilteritinib (gilt), or MRX-2843 (MRX), alone or in combination, for 24 h, and then plated in methylcellulose. The number of leukemic colonies (AML-CFUs), erythroid, and myeloid colonies were counted 10–14 days later. Data are presented as mean ± SEM. * indicates *p* < 0.05, ** indicates *p* < 0.01, and *** indicates *p* < 0.001 compared to control. ## indicates *p* < 0.01 and ### indicates *p* < 0.001 compared to single-drug treatments. Technical triplicates were performed.

To determine the effect of combined AZD5991 and gilteritinib or MRX-2843 on AML progenitor cells, we performed colony-forming assays. Three primary AML patient samples with *FLT3* mutations showed a significant decrease in their colony-forming capacity following the combined treatment with AZD5991 and gilteritinib or MRX-2843 compared to the single-drug treatment and vehicle control (Figure 2A). When normal human umbilical cord blood cells were tested, the gilteritinib treatment alone significantly reduced burst forming unit-erythroid colonies in one of the two samples tested. However, there was no further decrease when combined with AZD5991 (Figure 2B, left panel). The drug treatments did not show a significant negative effect on the colony-forming capacity of the other sample (Figure 2B, right panel). Taken together, these results demonstrate that AZD5991 and gilteritinib or MRX-2843 cooperate in suppressing AML progenitor cells but spare normal hematopoietic progenitors. 

### 3.3. c-Myc Plays an Important Role in the Synergistic Antileukemic Activity of AZD5991 and Gilteritinib or MRX-2843 in FLT3-Mutated AML Cells

**Figure 3:**
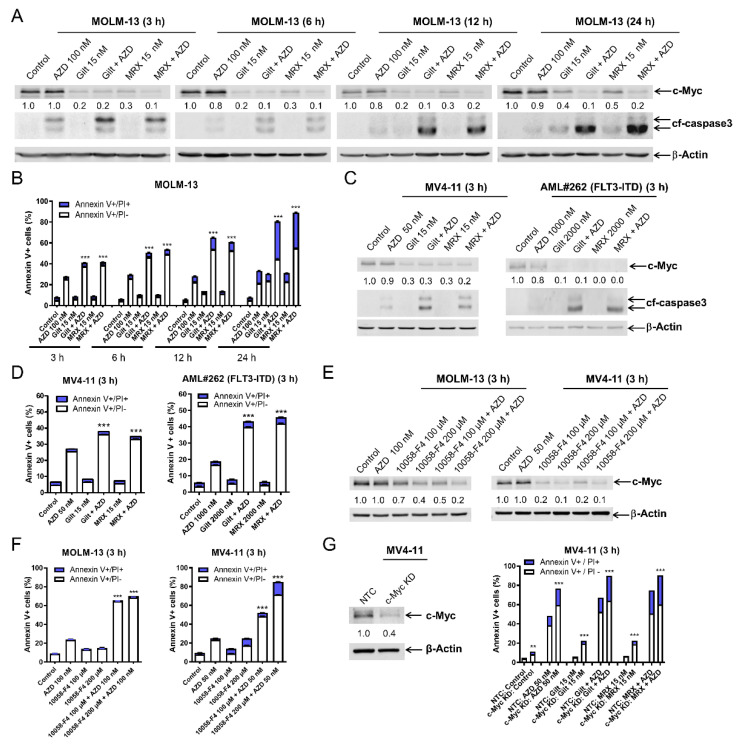
c-Myc plays an important role in the synergistic antileukemic activity of AZD5991 and gilteritinib or MRX-2843 in *FLT3*-mutated AML cells. (**A**) MOLM-13 cells were treated with AZD5991 (AZD), gilteritinib (gilt), or MRX-2843 (MRX), alone or in combination, for up to 24 h. Whole cell lysates were subjected to western blotting and probed with the indicated antibodies. Densitometry results (normalized to β-actin and compared to vehicle control at the matching time point) are shown below the corresponding blot. (**B**) MOLM-13 cells treated with AZD, gilt, or MRX, alone in combination, for up to 24 h, were stained with annexin V-FITC/PI and analyzed using a flow cytometer. *** indicates *p* < 0.001 compared to control and single-drug treatments. (**C**,**D**) MV4-11 and primary AML patient sample AML#262 cells were treated with AZD, gilt, MRX, or in combination for 3 h. Whole cell lysates were subjected to western blot analysis. Densitometry results (normalized to β-actin and compared to vehicle control) are shown below the corresponding blot in panel (**C**). After treatment, cells were stained with annexin V-FITC/PI and analyzed using a flow cytometer (panel (**D**)). *** indicates *p* < 0.001 compared to control and single-drug treatments. (**E**,**F**) MV4-11 and MOLM-13 cells were treated with vehicle control, AZD, 10058-F4, or AZD + 10058-F4 for 3 h. Whole cell lysates were subjected to western blotting analysis. Densitometry results (normalized to β-actin and compared to vehicle control) are shown below the corresponding blot (panel (**E**)). After treatment, cells were stained with annexin V-FITC/PI and analyzed using a flow cytometer (panel (**F**)). *** indicates *p* < 0.001 compared to control and single-drug treatments. (**G**) Lentiviral CRISPR/Cas9 knockdown (KD) of c-Myc was performed in MV4-11 cells along with non-target control (NTC). Whole cell lysates were subjected to western blotting (**left** panel). Cells were treated with vehicle control, AZD, gilt, MRX, or in combination for 3 h. Annexin V/PI staining and flow cytometry analysis results are shown in the (**right** panel). ** indicates *p* < 0.01 and *** *p* < 0.001 compared to NTC for the same drug treatment.

To begin to determine the mechanism of action of the combinations, we performed time course experiments. Western blot analysis of MOLM-13 cells revealed a downregulation of c-Myc protein by gilteritinib and MRX-2843 single treatments as early as 3 h (Figure 3A), which was prior to the significant induction of apoptosis by these single-drug treatments (Figure 3B). Gilteritinib and MRX-2843’s downregulation of c-Myc was maintained when AZD5991 was added and was accompanied by a significant induction of apoptosis compared to the single-drug treatment as early as 3 h. Similar results were obtained when MV4-11 and primary AML patient AML#262 cells were treated for 3 h (Figure 3C,D). The c-Myc inhibitor 10058-F4 downregulated c-Myc and enhanced the apoptosis induced by AZD5991 in both the MV4-11 and MOLM-13 cells (Figure 3E,F). Further, c-Myc knockdown significantly enhanced apoptosis induced by AZD5991, gilteritinib, and MRX-2843, alone and in combination, in the MV4-11 cells (Figure 3G). These results demonstrate that c-Myc plays an important role in the synergistic antileukemic activity of AZD5991 and gilteritinib or MRX-2843 in AML cells. 

### 3.4. FLT3 Inhibition Decreases c-Myc Protein Level via Transcriptional Regulation through Suppression of the MEK/ERK and JAK2/STAT5 Pathways

**Figure 4:**
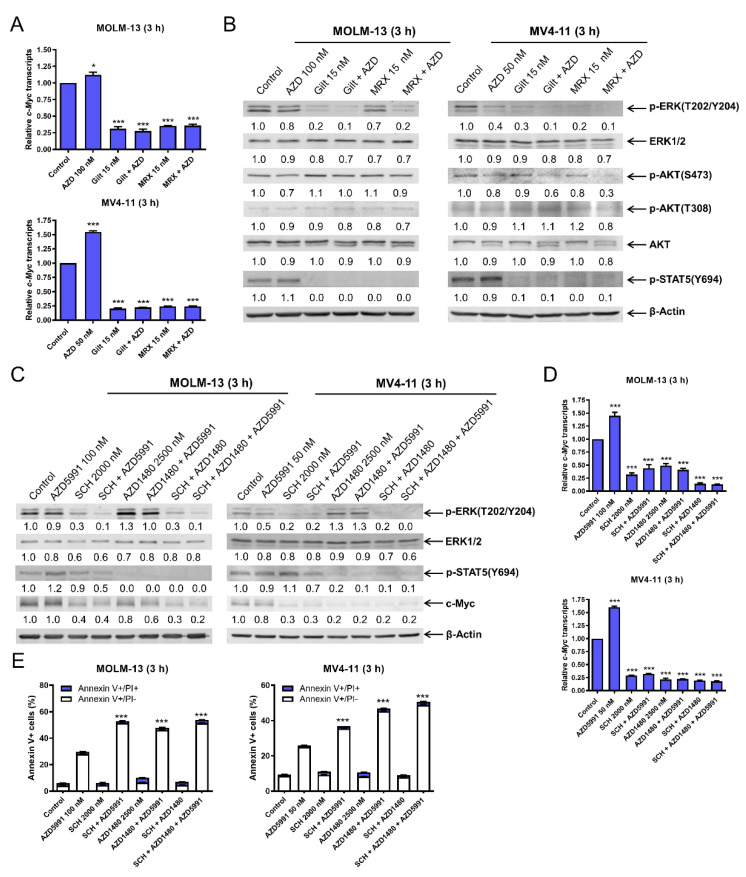
FLT3 inhibition decreases c-Myc protein via transcriptional regulation through suppression of the MEK/ERK and JAK2/STAT5 pathways. (**A**) MOLM-13 and MV4-11 cells were treated with AZD5991 (AZD), gilteritinib (gilt), or MRX-2843 (MRX), alone or in combination, for 3 h. Total RNA was extracted, and real-time RT-PCR was performed. The displayed results represent the mean of three independent experiments, with fold changes calculated via comparative Ct method and normalized to *36B4* transcripts. * indicates *p* < 0.05 and *** indicates *p* < 0.001 compared to the vehicle control. (**B**) MOLM-13 and MV4-11 cells were treated with vehicle control, AZD, gilt, MRX, or in combination for 3 h. Western blots were generated utilizing whole cell lysates. Densitometry results (normalized to β-actin and compared to vehicle control) are shown below the corresponding blot. (**C**–**E**) MOLM-13 and MV4-11 cells were treated with vehicle control, AZD, SCH772984 (SCH), or AZD1480, alone or in combination, for 3 h. Whole cell lysates were subjected to western blotting analysis. Representative western blots are shown in panel (**C**). Densitometry results (normalized to β-actin and compared to vehicle control) are shown below the corresponding blot. After treatment, total RNA was extracted, and real-time RT-PCR was performed (panel (**D**)). The displayed results represent the mean of three independent experiments, with fold changes calculated via comparative Ct method and normalized to *36B4* transcripts. *** indicates *p* < 0.001 compared to vehicle control. Treated cells were stained with annexin V-FITC/PI and analyzed using a flow cytometer (panel (**E**)). *** indicates *p* < 0.001 compared to control and single-drug treatments.

To understand how FLT3 inhibition downregulates c-Myc, we determined the effect of FLT3 inhibition on *c-Myc* transcript levels. AZD5991 treatment alone resulted in a significant increase in *c-Myc* transcript levels, while gilteritinib or MRX-2843 treatment alone or in combination with AZD5991 significantly decreased *c-Myc* transcript levels (Figure 4A). In contrast, the c-Myc protein half-life was not significantly shortened by the combined treatments (Figure S3), suggesting that the downregulation of c-Myc occurred via transcriptional regulation.

In our previous studies, we found that at early time points FLT3 inhibition decreases phosphorylated ERK (T202/Y204) and phosphorylated STAT5 (Y694) [14]. Consistent with those findings, both gilteritinib and MRX-2843 treatments for 3 h decreased phosphorylated ERK (T202/Y204) and phosphorylated STAT5 (Y694) (Figure 4B). To determine the role of ERK and JAK2/STAT5 in c-Myc downregulation and the apoptosis induced by FLT3 inhibition and AZD5991 treatment, alone or in combination, MOLM-13 and MV4-11 cells were treated with the selective ERK inhibitor SCH772984 (SCH) and the JAK1/2 inhibitor AZD1480 alone and combined, with and without AZD5991 (Mcl-1 inhibition) (Figure 4C). The inhibition of ERK with SCH decreased p-ERK, ERK, and c-Myc, and lower levels were maintained when Mcl-1 was also inhibited (AZD5991 plus SCH). The combined Mcl-1 and ERK inhibition with AZD5991 and SCH also decreased p-STAT5. The inhibition of JAK1/2 with AZD1480 reduced p-STAT5 and this reduction was maintained when combined with Mcl-1 inhibition (AZD5991 plus AZD1480). Additionally, combined Mcl-1 and JAK1/2 inhibition resulted in decreased c-Myc. When both ERK and JAK1/2 were inhibited via SCH and AZD1480 treatments, respectively, there were similar reductions of p-ERK and c-Myc as compared to SCH alone, and similar reductions of p-STAT5 as compared to AZD1480 treatment alone. Inhibiting Mcl-1, ERK, and JAK1/2 using the three-drug combination resulted in decreased levels of p-ERK (similar to SCH plus AZD5991), p-STAT5 (similar to AZD1480 alone), and c-Myc (similar to SCH plus AZD1480). ERK inhibition, alone and combined with Mcl-1 inhibition (SCH plus AZD5991), decreased *c-Myc* transcripts (Figure 4D). JAK1/2 inhibition, alone and in combination with Mcl-1 inhibition (AZD1480 plus AZD5991), also resulted in decreased *c-Myc* transcripts. Finally, both ERK inhibition via SCH treatment and JAK1/2 inhibition via AZD1480 treatment significantly enhanced AZD5991-induced apoptosis (Figure 4E). Taken together, these results demonstrate that the inactivation of the MEK/ERK and JAK2/STAT5 pathways via FLT3 inhibition plays a role in the transcriptional downregulation of c-Myc and the synergistic induction of apoptosis by combined FLT3 inhibition and Mcl-1 inhibition in *FLT3*-mutated AML cells. 

### 3.5. FLT3 Inhibition Enhances AZD5991-Induced Cytochrome c Release

**Figure 5:**
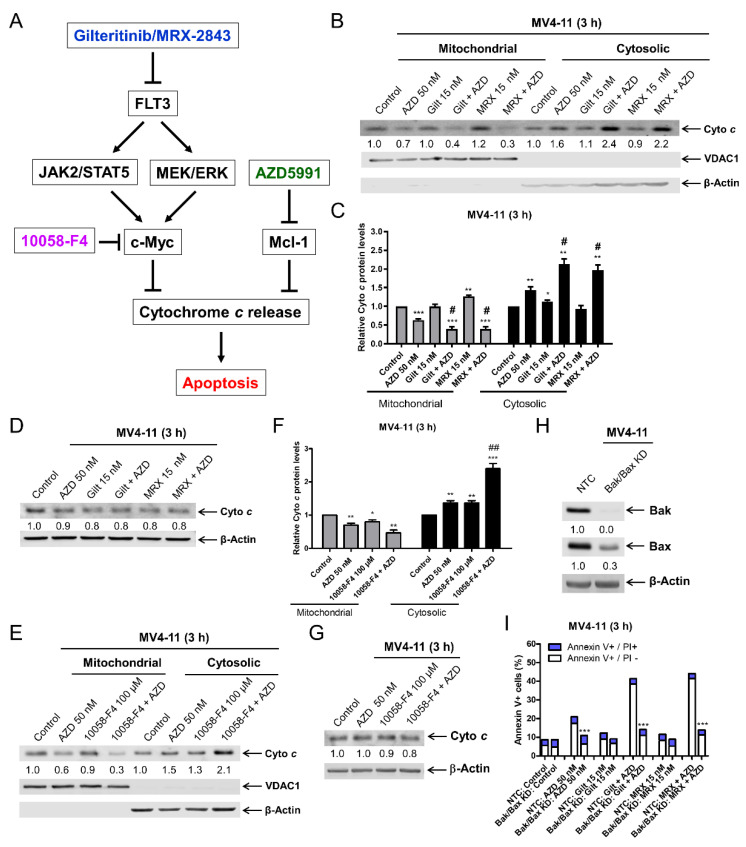
FLT3 inhibition enhances AZD5991-induced cytochrome *c* release. (**A**) Proposed mechanism of action of gilteritinib/MXR-2843 in combination with AZD5991. (**B**,**C**) MV4-11 cells were treated with vehicle control, AZD5991 (AZD), gilteritinib (gilt), MRX-2843 (MRX), or in combination for 3 h. Cellular fractionation was performed. Mitochondrial and cytosolic fractions were subjected to western blotting analysis. This experiment was performed two independent times in triplicate. One representative image is shown. Relative densitometry measurements were determined, normalized to β-actin or VDAC1, and compared to the vehicle control. Results from one representative experiment are graphed as mean ± SEM in panel (**C**). * indicates *p* < 0.05, ** indicates *p* < 0.01, and *** indicates *p* < 0.001 compared to vehicle control. # indicates *p* < 0.05 compared to single-drug treatment. (**D**) MV4-11 cells were treated with vehicle control, AZD, gilt, MRX, or in combination for 3 h. Western blots were generated utilizing whole cell lysates. Densitometry results (normalized to β-actin and compared to vehicle control) are shown below the corresponding blot. (**E**,**F**) MV4-11 cells were treated with vehicle control, AZD, 10058-F4, or in combination for 3 h. Cellular fractionation was performed as described in panel (**B**). * indicates *p* < 0.05, ** indicates *p* < 0.01 and *** indicates *p* < 0.001 compared to vehicle control. ## indicates *p* < 0.01 compared to single-drug treatment. (**G**) MV4-11 cells were treated with vehicle control, AZD, 10058-F4, or in combination for 3 h. Western blots were generated utilizing whole cell lysates. Densitometry results (normalized to β-actin and compared to vehicle control) are shown below the corresponding blot. (**H**,**I**) shRNA knockdown of Bak and Bax was performed in MV4-11 cells with non-template control (NTC) as the negative control. Whole cell lysates were subjected to western blotting (panel (**H**)). Cells were treated with vehicle control, AZD, gilt, MRX, or in combination for 3 h. Annexin V/PI staining and flow cytometry analysis results are shown in panel (**I**). *** *p* < 0.001 compared to NTC for the same drug treatment.

c-Myc has been shown to regulate cytochrome *c* release [36]. Based on this, we hypothesized that a downregulation of c-Myc by FLT3 inhibition would increase the cytochrome *c* release induced by AZD5991, leading to enhanced apoptosis (Figure 5A). The inhibition of FLT3 with gilteritinib alone and MRX-2843 alone had little to no effect on the subcellular localization of cytochrome *c*, while AZD5991 treatment alone for 3 h significantly increased cytochrome *c* in the cytosolic fraction and significantly decreased cytochrome *c* in the mitochondrial fraction in the MV4-11 cells (Figure 5B,C). The combination treatment resulted in a further significant increase in cytochrome *c* in the cytosolic fraction and a decrease in the mitochondrial fraction. The total cytochrome *c* levels remained largely unchanged (Figure 5D). To compliment this, the treatment with the c-Myc inhibitor 10058-F4 alone also significantly increased cytochrome *c* release, which was further significantly increased when combined with AZD5991 (Figure 5E–G). Similar results were obtained in MOLM-13 cells (Figure S4). A Bak/Bax double knockdown model was utilized to investigate the activation of the intrinsic apoptosis pathway by the combinations. The Bak/Bax double knockdown almost completely rescued MV4-11 cells from AZD5991, gilteritinib + AZD5991, and MRX-2843 + AZD5991-induced apoptosis (Figure 5I). Taken together, these results demonstrate that FLT3 inhibition enhances AZD5991-induced cytochrome *c* release and subsequent apoptosis. 

### 3.6. AZD5991 Synergistically Enhances Apoptosis Induced by Gilteritinib and MRX-2843 in AraC Resistant AML Cells

**Figure 6:**
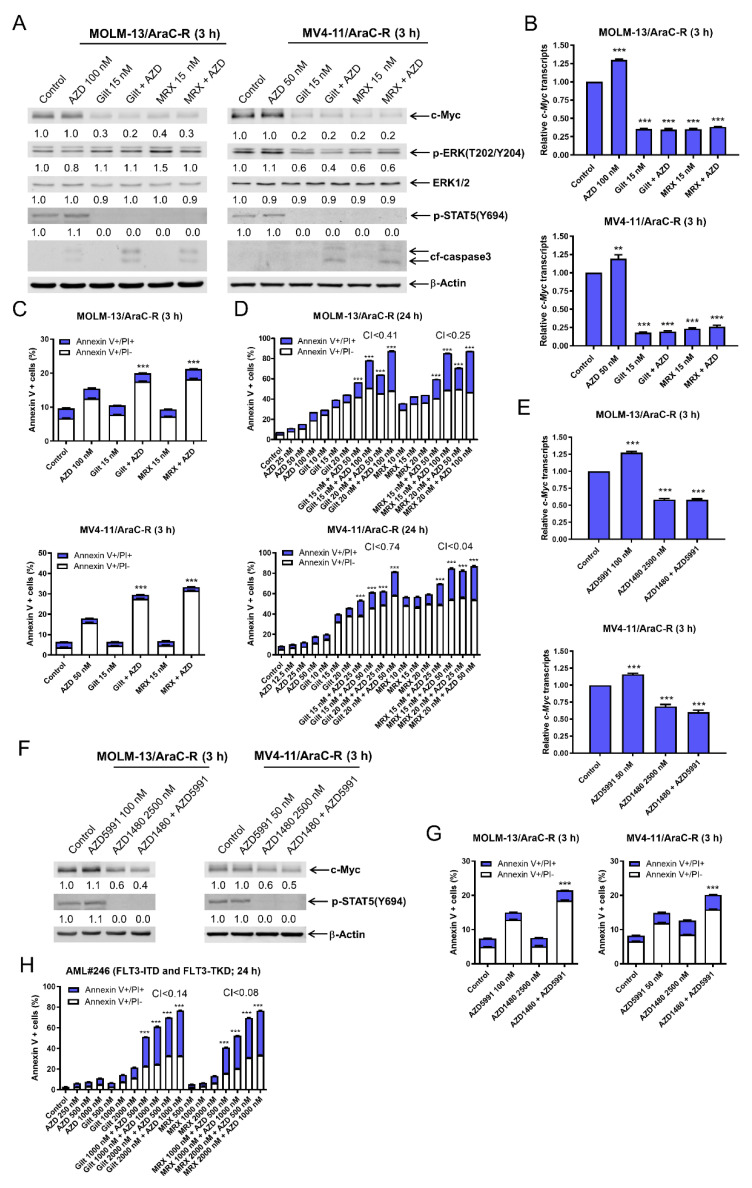
AZD5991 synergistically enhances apoptosis induced by gilteritinib and MRX-2843 in AraC-resistant AML cells. (**A**) MOLM-13 and MV4-11 AraC-resistant cells (MOLM-13/AraC-R and MV4-11/AraC-R) were treated with vehicle control, AZD5991 (AZD), gilteritinib (gilt), MRX-2843 (MRX), or in combination for 3 h. Whole cell lysates were subjected to Western blotting. Densitometry results (normalized to β-actin and compared to vehicle control) are shown below the corresponding blot. (**B**) MOLM-13/AraC-R and MV4-11/AraC-R cells were treated with vehicle control, AZD, gilt, MRX, or in combination for 3 h. Total RNA was extracted, and real-time RT-PCR was performed. The displayed results represent the mean of three independent experiments, with fold changes calculated via comparative Ct method and normalized to *36B4* transcripts. ** indicates *p* < 0.01 and *** indicates *p* < 0.001 compared to vehicle control. (**C**) MOLM-13/AraC-R and MV4-11/AraC-R cells were treated with vehicle control, AZD, gilt, MRX, or in combination for 3 h. Annexin V/PI staining and flow cytometry analysis results are shown. *** indicates *p* < 0.001 compared to vehicle control and single-drug treatment. (**D**) MOLM-13/AraC-R and MV4-11/AraC-R cells were treated with vehicle control, AZD, gilt, MRX, or in combination for 24 h. Annexin V/PI staining and flow cytometry analysis results are shown. *** indicates *p* < 0.001 compared to vehicle control and single-drug treatment. CI values were calculated using CompuSyn software. CI < 1, CI = 1, and CI > 1 indicate synergistic, additive, and antagonistic effects, respectively. (**E**) MOLM-13/AraC-R and MV4-11/AraC-R cells were treated with vehicle control, AZD, gilt, MRX, or in combination for 3 h. Total RNA was extracted, and real-time RT-PCR was performed. The displayed results represent the mean of three independent experiments, with fold changes calculated via comparative Ct method and normalized to *36B4* transcripts. *** indicates *p* < 0.001 compared to control and single-drug treatments. (**F**,**G**) MOLM-13/AraC-R and MV4-11/AraC-R cells were treated with vehicle control, AZD5991 (AZD), AZD1480, or in combination for 3 h. Whole cell lysates were subjected to western blotting. Densitometry results (normalized to β-actin and compared to vehicle control) are shown below the corresponding blot in panel (**F**). After treatment, cells were stained with annexin V-FITC/PI and analyzed using a flow cytometer (panel (**G**)). *** indicates *p* < 0.001 compared to control and single-drug treatments. (**H**) Primary AML patient sample AML#246 cells treated with varying concentrations of AZD, gilt, or MRX, alone or in combination, for 24 h were stained with annexin V-FITC/PI and analyzed using a flow cytometer. CI values were calculated using CompuSyn software. *** indicates *p* < 0.001 compared to vehicle and single-drug treatments.

Gilteritinib is FDA-approved for the treatment of relapsed/refractory patients with *FLT3*-mutated AML. Thus, it is important to test if the combination of AZD5991 and FLT3 inhibition is effective against chemotherapy-resistant AML cells. MOLM-13 and MV4-11 cells with acquired AraC resistance (designated MOLM-13/AraC-R and MV4-11-R, respectively) were previously generated and demonstrate increased levels of c-Myc when compared to the parental cells [14,15]. Consistent with the parental cells, the AraC-resistant cells also showed a downregulation of c-Myc and p-STAT5 after a 3 h treatment with gilteritinib or MRX-2843 alone or in combination with AZD5991 (Figure 6A). The downregulation of p-ERK was detected in the MV4-11/AraC-R cells after the treatment with MRX-2843 or gilteritinib alone or in combination with AZD5991, but not in the MOLM-13/AraC-R cells. The downregulation of the c-Myc protein by gilteritinib or MRX-2843 both alone and in combination with AZD5991 was accompanied by significantly decreased *c-Myc* transcript levels (Figure 6B). Consistent with the parental cells, AZD5991 treatment alone resulted in a significant increase in *c-Myc* transcript levels. AZD5991 treatment significantly enhanced gilteritinib and MRX-2843-induced apoptosis at this early treatment time (Figure 6C). The treatment with AZD5991 combined with gilteritinib or MRX-2843 for 24 h resulted in synergistic induction of apoptosis (Figure 6D). The treatment with the JAK2 inhibitor, AZD1480, alone or combined with AZD5991 significantly decreased *c-Myc* transcript levels in the AraC-R cells (Figure 6E), which was accompanied by substantially reduced c-Myc protein levels (Figure 6F). Consistent with the parental cells, the AZD1480 (JAK1/2 inhibitor) and AZD5991 (Mcl-1 inhibitor) treatment significantly induced apoptosis in the AraC-R cells after 3 h of treatment (Figure 6G). The combination of AZD5991 and gilterinib or MRX-2843 also synergistically induced apoptosis in the primary AML cells derived from a patient with *FLT3*-ITD and -TKD AML who failed to achieve remission after induction 3 chemotherapy (Figure 6H). These results demonstrate that the combination of Mcl-1 inhibition and FLT3 inhibition shows promising antileukemic activities against AML cells resistant to chemotherapy, and that the suppression of c-Myc through the JAK2/STAT5 pathway by FLT3 inhibition plays an important role in the mechanism of action of combined Mcl-1 inhibition with AZD5991 and FLT3 inhibition with gilteritinib or MRX-2843.

## 4. Discussion

FLT3 inhibitors have promise in treating *FLT3*-mutated AML; however, the responses are short-lived, highlighting the need to develop combination therapies to enhance the antileukemic activities of these agents. Recently, we reported that the suppression of c-Myc plays a critical role in the antileukemic activity of the Mcl-1 inhibitor AZD5991 [15] and the downregulation of c-Myc post-FLT3 inhibitor treatment [14]. In addition, FLT3 inhibitors have been shown to downregulate Mcl-1 [11,12] and gilteritinib treatment substantially decreases Mcl-1 protein levels in AML cells [12,14]. These findings prompted us to investigate the combination of AZD5991 (as a prototype Mcl-1 inhibitor) and the FLT3 inhibitors gilteritinib and MRX-2843. The combinations synergistically induced apoptosis in the *FLT3*-mutated AML cell lines and primary patient samples (Figure 1). These results are consistent with Singh Mali et al., who found that the Mcl-1 inhibitor AMG 176 improved the survival of NSG mice inoculated with MV4-11 cells when combined with the FLT3 inhibitor quizartinib [37]. Interestingly, we found that AZD5991 and gilteritinib or MRX-2843 also synergize in inducing apoptosis in *FLT3*-wt AML cell lines and primary patient samples (Figures S1 and S2), which is likely due to the expression of wt-FLT3, AXL and/or MERTK in these cells (Figure S1). Moreover, AZD5991 and gilteritinib or MRX-2843 cooperated in the suppression of the *FLT3*-mutated AML progenitor cells but spared normal hematopoietic progenitor cells, indicating a therapeutic window for these promising combination therapies. However, in vivo studies are needed to determine the efficacy and tolerability of the combinations of AZD5991 with gilteritinib or MRX-2843.

Consistent with our previous studies [12,14], gilteritinib and MRX-2843 suppressed Mcl-1 in the FLT3-mutated AML cells (Figure S5). As we have demonstrated in our most recent study that the downregulation of Mcl-1 significantly enhances apoptosis induced by AZD5991 [15], we focused on the role c-Myc plays in the synergy between AZD5991 and gilteritinib or MRX-2843. The CRISPR knockdown and pharmacological inhibition of c-Myc significantly enhanced the apoptosis induced by AZD5991 (Figure 3E–G), demonstrating that c-Myc plays an important role in the synergy between AZD5991 and FLT3 inhibition. The downregulation of c-Myc by FLT3 inhibitors was determined to be caused through transcriptional mechanisms as the FLT3 inhibitors significantly decreased *c-Myc* transcripts but had no effect on c-Myc protein stability (Figure 4A and Figure S3). This transcriptional suppression of c-Myc by the FLT3 inhibitors is likely mediated by the suppression of ERK and STAT5 (Figure 4B–E), though ERK inactivation appeared to be cell line-dependent in the AraC-resistant AML cell lines (Figure 6). However, the precise molecular mechanism still needs to be determined, which is beyond the scope of this study.

As an antiapoptotic member of the Bcl-2 family, Mcl-1 plays a critical role in the intrinsic apoptosis pathway. Consistent with this, the inhibition of Mcl-1 via AZD5991 results in intrinsic apoptosis in AML cells as demonstrated in our most recent study [15]. Cytochrome *c* is a critical mediator of intrinsic apoptosis, and a major portion of cytochrome *c* is bound to cardiolipin, tethering it to the mitochondrial inner membrane [38,39]. There is evidence to suggest that this interaction limits the release of cytochrome *c* [40]. However, ROS can readily oxidize cardiolipin, resulting in a greater portion of untethered cytochrome *c* in the intermembrane space. To further support the increased release of cytochrome *c* in AML cells following the treatment with AZD5991 in combination with FLT3 inhibitors, we also found increased mitochondrial ROS in the *FLT3*-mutated AML cells post AZD5991 and FLT3 inhibitor treatments (Figure S6). An overexpression of Mcl-1 in AML cells has been reported to increase mitochondrial ROS [41], and we have previously demonstrated that the treatment of AML cells with AZD5991 results in increased Mcl-1 protein, which may explain these results. The c-Myc inhibitor 10058-F4 also induced a significant increase in mitochondrial ROS, though the treatment with gilteritinib or MRX-2843 alone had no significant effect on mitochondrial ROS levels. The combination treatment with AZD5991 and gilteritinib, MRX-2843, or 10058-F4 resulted in a further significant increase in mitochondrial ROS. Thus, it is possible that the combined inhibition of Mcl-1 and suppression of c-Myc increases mitochondrial ROS, oxidizing cardiolipin, the untethering of a portion of cytochrome *c* in the mitochondrial intermembrane space, and results in greater cytochrome *c* release. It is unclear why gilteritinib or MRX-2843 treatment alone did not increase mitochondrial ROS, but we speculate that it is due to the multiple pathways affected by FLT3 inhibition as opposed to c-Myc inhibition alone.

c-Myc plays an important role in cellular metabolism [42] and Mcl-1 is similarly important in mitochondrial metabolism [43] and apoptosis. As such, drug-induced changes to cellular metabolism may play a role in the synergy. Interestingly, we have reported that the mitochondrial complex I inhibitor IACS-010759 induces a vulnerable mitochondrial state that sensitizes AML cells to venetoclax and cytochrome *c* release [29]. In this study, we have similarly found that FLT3 inhibition enhances AZD5991-induced cytochrome *c* release. Given that gilteritinib treatment has been reported to decrease the oxygen consumption rate in *FLT3*-ITD AML cells, indicating a reduction in oxidative phosphorylation [44], this may also play a role in the mechanism of action, though further investigation into this mechanism of action is beyond the scope of this manuscript.

Finally, it is well known that c-Myc plays a pivotal role in the regulation of proliferation, differentiation, and apoptosis in myeloid cells and that its expression is significantly higher in AML patients compared to healthy controls. [45,46] c-Myc expression is correlated with a poor overall survival in intermediate and favorable cytogenetic risk groups. [47] Additionally, c-Myc is correlated with chemoresistance in AML patients and targeting both c-Myc and the downstream factors regulated by c-Myc suppresses this resistance. [48,49,50,51] We show here that the inhibition of Mcl-1 via AZD5991 significantly enhances cell death induction by FLT-3 inhibitors and the combinations remarkably reduced both protein and transcript levels of c-Myc in AraC-resistant cell lines. Similarly, we demonstrate the significant induction of cell death and synergy between these Mcl-1 and FLT-3 inhibitors in treatment-resistant primary patient cells. These results demonstrate that the combinations of Mcl-1 inhibition and FLT3 inhibition show promising antileukemic activities against AML cells resistant to chemotherapy.

## 5. Conclusions

In summary, the inhibition of Mcl-1 via AZD5991 synergizes with FLT3 inhibition against AML cells. In vitro, the combinations are selective for AML cells compared with normal hematopoietic progenitor cells, suggesting a therapeutic window. Both gilteritinib and MRX-2843 in combination with AZD5991 show synergistic antileukemic activity against AraC-resistant *FLT3*-mutated AML cells. Our findings provide support for the development of the Mcl-1 inhibitors in combination with FLT3 inhibitors for the treatment of AML, including those with acquired resistance to AraC.

## Data Availability

All data generated or analyzed for this study are included in this published article or are available upon request to either Yubin Ge (gey@karmanos.org) or Guan Wang (wg10@jlu.edu.cn).

## Supplementary Materials

The following supporting information can be downloaded at: https://www.mdpi.com/article/10.3390/cells11172752/s1, Figure S1, AZD5991 synergistically enhances apoptosis induced by gilteritinib and MRX-2843 in *FLT3*-wt AML cell lines; Figure S2, AZD5991 synergistically enhances apoptosis induced by gilteritinib and MRX-2843 in *FLT3*-wt AML patient samples; Figure S3, Protein stability does not likely play a role in the suppression of c-Myc by gilteritinib and MRX-2843; Figure S4, FLT3 inhibition enhances AZD5991-induced cytochrome *c* release in MOLM-13 cells; Figure S5, Gilteritinib and MRX-2843 suppress Mcl-1 in *FLT3*-mutated AML cells; Figure S6, Gilteritinib and MRX-2843 enhance AZD5991-induced mitochondrial ROS in *FLT3*-ITD AML cells; Table S1: Patient characteristics of the primary AML patient samples used in this study.
