# Supplementary material for: Inhibition of Mcl-1 Synergistically Enhances the Antileukemic Activity of Gilteritinib and MRX-2843 in Preclinical Models of FLT3-Mutated Acute Myeloid Leukemia
Source: Cells. 2022 Sep 3;11(17):2752. doi: 10.3390/cells11172752 (PMC9455003; doi:10.3390/cells11172752)
Supplement: Supplementary file 1 [file cells-11-02752-s001.zip › cells-1876944-supplementary.pdf]

Inhibition of Mcl-1 Synergistically Enhances the Antileukemic Activity of Gilteritinib and MRX-2843 in Preclinical Models of *FLT3*-mutated Acute Myeloid Leukemia

Shuangshuang Wu [1,2], Holly Edwards [3,4], Deying Wang [5], Shuang Liu [2], Xinan Qiao [2], Jenna Carter [6,7], Yue Wang [1], Jeffrey W. Taub [8,9,10], Guan Wang [2,*], and Yubin Ge [3,4,6,*]
#

[1] Department of Pediatric Hematology, The First Hospital of Jilin University, Changchun 130021, China

[2] National Engineering Laboratory for AIDS Vaccine, School of Life Sciences, Jilin University, Changchun 130012, China

[3] Department of Oncology, Wayne State University School of Medicine, Detroit, MI 48202, USA

[4] Molecular Therapeutics Program, Karmanos Cancer Institute, Wayne State University School of Medicine, Detroit, MI 48201, USA

[5] The Tumor Center of the First Hospital of Jilin University, Changchun 130021, China

[6] Cancer Biology Graduate Program, Wayne State University School of Medicine, Detroit, MI 48201, USA

[7] MD/PhD Program, Wayne State University School of Medicine, Detroit, MI 48201, USA

[8] Department of Pediatrics, Wayne State University School of Medicine, Detroit, MI 48202, USA

[9] Division of Pediatric Hematology and Oncology, Department of Pediatrics, Children's Hospital of Michigan, Detroit, MI 48201, USA

[10] Department of Pediatrics, Central Michigan University College of Medicine, Mt. Pleasant, MI 48859, USA

[*]Correspondence: gey@karmanos.org; Tel:313-578-4285 (Y.G.); wg10@jlu.edu.cn (G.W.)

Materials

MitoSOX™ Red was purchased from Thermo Fisher Scientific (Rockford, IL, USA). Cycloheximide was purchased from Sigma-Aldrich (St. Louis, MO, USA).

Methods

Detection of mitochondrial reactive oxygen species (ROS)

MV4-11 and MOLM-13 cells were treated with the indicated drugs for 3 hours. The treated cells were washed with PBS twice and resuspended in PBS (3×10^5 cells/0.5 mL), and 0.5 μL of 5 mM MitoSOX Red solution was added to the cells and incubated at 37°C for 10 minutes in the dark. Then the cells were washed with PBS three times, and mean fluorescence intensity was detected by flow cytometry and normalized to vehicle control.

Legends for supplemental figures

Figure S1. AZD5991 synergistically enhances apoptosis induced by gilteritinib and MRX-2843 in *FLT3*-wt AML cell lines. (A) Whole cell lysates from HL-60, MOLM-13, MV4-11, OCI-AML3, and THP-1 cells were subjected to western blotting and probed with the indicated antibodies. Densitometry results (normalized to β-actin and compared to MOLM-13 or OCI-AML3) are shown below the corresponding blot. (B) HL-60, OCI-AML3, and THP-1 cells treated with varying concentrations of AZD5991 (AZD), gilteritinib (gilt), or MRX-2843 (MRX), alone or in combination, for 24 h were stained with annexin V-FITC/propidium iodide (PI) and analyzed using a flow cytometer. Combination Index (CI) values were calculated using CompuSyn software. CI < 1, CI = 1, and CI > 1 indicate synergistic, additive, and antagonistic effects, respectively. *** indicates $p < 0.001$. (C) THP-1 cells were treated with AZD, gilt, AZD + gilt, MRX, or MRX +

AZD for 24 h. Whole cell lysates were subjected to western blotting analysis and probed with the indicated antibodies.

Figure S2. AZD5991 synergistically enhances apoptosis induced by gilteritinib and MRX-2843 in *FLT3*-wt primary AML patient samples. Primary AML patient sample cells treated with varying concentrations of AZD5991 (AZD), gilteritinib (gilt), or MRX-2843 (MRX), alone or in combination, for 24 h were stained with annexin V-FITC/PI and analyzed using a flow cytometer. CI values were calculated using CompuSyn software. CI < 1, CI = 1, and CI > 1 indicate synergistic, additive, and antagonistic effects, respectively. *** indicates $p < 0.001$. ND indicates not determined.

Figure S3. Protein stability does not likely play a role in the suppression of c-Myc by gilteritinib and MRX-2843. (A) MOLM-13 cells were treated with vehicle control, AZD, gilt, MRX, or in combination for 3 h. The cells were washed and then treated with 10 μg/mL cycloheximide for up to 2 h. Western blots were generated utilizing whole cell lysates. Representative blots are shown in panel A. Densitometry measurements (normalized to β-actin and compared to 0 min) are graphed in panel B. c-Myc half-life was calculated using GraphPad Prism (panel C).

Figure S4. FLT3 inhibition enhances AZD5991-induced cytochrome *c* release in MOLM-13 cells. MOLM-13 cells were treated with vehicle control, AZD5991 (AZD), gilteritinib (gilt), MRX-2842 (MRX), or in combination for 3 h. Cellular fractionation was performed. Mitochondrial and cytosolic fractions were subjected to western blot analysis. This experiment

was performed 2 independent times in triplicate. One representative image is shown in panel A. Relative densitometry measurements were determined, normalized to β-actin or VDAC1, and compared to the vehicle control. Results from one representative experiment are graphed as mean ± SEM in panel B. * indicates $p < 0.05$, ** indicates $p < 0.01$ and *** indicates $p < 0.001$ compared to vehicle control. # indicates $p < 0.05$ compared to single drug treatment. (C)#MOLM-13 cells were treated vehicle control, AZD, gilt, MRX, or in combination for 3 h. Western blots were generated utilizing whole cell lysates. Densitometry results (normalized to β-actin and compared to vehicle control) are shown below the corresponding blot. (D&E) MOLM-13 cells were treated with vehicle control, AZD, 10058-F4, or in combination for 3 h. Cellular fractionation was performed. Mitochondrial and cytosolic fractions were subjected to western blot analysis. This experiment was performed 2 independent times in triplicate. One representative image is shown in panel D. Relative densitometry measurements were determined, normalized to β-actin or VDAC1, and compared to the vehicle control. Results from one representative experiment are graphed as mean ± SEM in panel E. * indicates $p < 0.05$, ** indicates $p < 0.01$ and *** indicates $p < 0.001$ compared to vehicle control. ### indicates $p < 0.001$ compared to single drug treatment. (F) MOLM-13 cells were treated with vehicle control, AZD, 10058-F4, or in combination for 3 h. Western blots were generated utilizing whole cell lysates. Densitometry results (normalized to β-actin and compared to vehicle control) are shown below the corresponding blot.

Figure S5. Gilteritinib and MRX-2843 suppress Mcl-1 in *FLT3*-mutated AML cells. MOLM-13, MV4-11, and primary AML cells (AML#246) were treated with vehicle control, AZD5991 (AZD), gilteritinib (Gilt), or MRX-2843 (MRX), alone or in combination, for 3 h. Whole cell lysates were subjected to western blotting and probed with the indicated antibodies. Densitometry

results (normalized to β-actin and compared to vehicle control) are shown below the corresponding blot.

Figure S6. Gilteritinib and MRX-2843 enhance AZD5991-induced mitochondrial ROS in FLT3-ITD AML cells. (A) MV4-11 and MOLM-13 cells were treated with vehicle control, AZD5991 (AZD), gilteritinib (gilt), or MRX-2843 (MRX), alone or in combination, for 3 h. Cells were stained with MitoSOX Red and analyzed by flow cytometry. *** indicates $p < 0.001$ compared to control. ## indicates $p < 0.01$ and ### indicates $p < 0.001$ compared to single drug treatment. (B) MV4-11 and MOLM-13 cells were treated with vehicle control, AZD, 10058-F4, or AZD + 10058-F4 for 3 h. Cells were stained with MitoSOX Red and analyzed by flow cytometry. *** indicates $p < 0.001$ compared to control. ## indicates $p < 0.01$ and ### indicates $p < 0.001$ compared to single drug treatment.

Figure S1

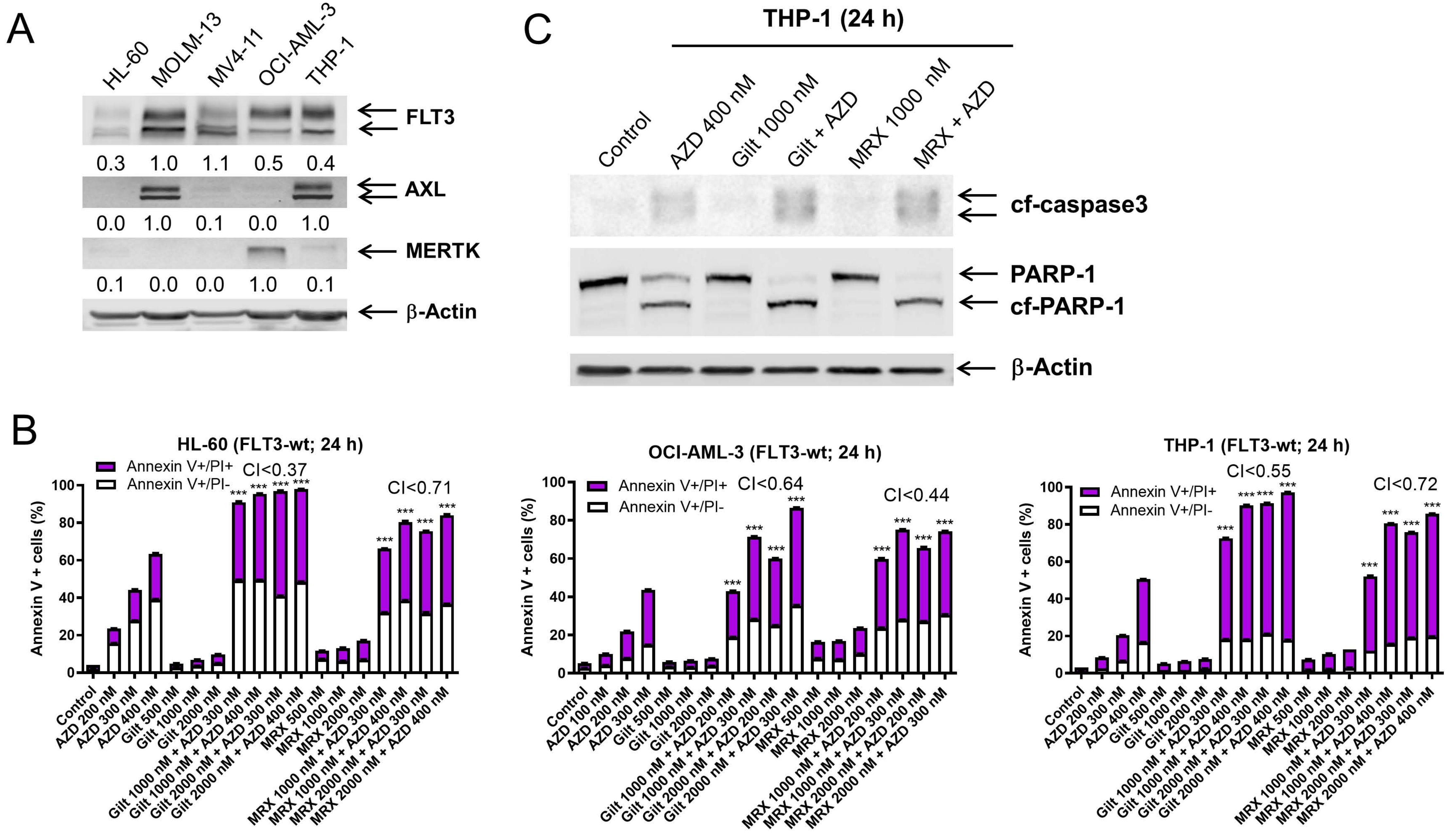

A
HL-60
MOLM-13
MV4-11
OCI-AML-3
THP-1
FLT3
0.3 1.0 1.1 0.5 0.4
AXL
0.0 1.0 0.1 0.0 1.0
MERTK
0.1 0.0 0.0 1.0 0.1
β-Actin
C
THP-1 (24 h)
Control
AZD 400 nM
Gilt 1000 nM
Gilt + AZD
MRX 1000 nM
MRX + AZD
cf-caspase3
PARP-1
cf-PARP-1
β-Actin
B
HL-60 (FLT3-wt; 24 h)
Annexin V+/PI+
Annexin V+/PI-
CI<0.37
CI<0.71
Annexin V + cells (%)
Control
AZD 200 nM
AZD 300 nM
AZD 400 nM
Gilt 500 nM
Gilt 1000 nM
Gilt 2000 nM
Gilt 1000 nM + AZD 300 nM
Gilt 1000 nM + AZD 400 nM
Gilt 2000 nM + AZD 300 nM
Gilt 2000 nM + AZD 400 nM
MRX 500 nM
MRX 1000 nM
MRX 2000 nM
MRX 1000 nM + AZD 300 nM
MRX 1000 nM + AZD 400 nM
MRX 2000 nM + AZD 300 nM
MRX 2000 nM + AZD 400 nM
OCI-AML-3 (FLT3-wt; 24 h)
Annexin V+/PI+
Annexin V+/PI-
CI<0.64
CI<0.44
Annexin V + cells (%)
Control
AZD 100 nM
AZD 200 nM
AZD 300 nM
Gilt 500 nM
Gilt 1000 nM
Gilt 2000 nM
Gilt 1000 nM + AZD 200 nM
Gilt 1000 nM + AZD 300 nM
Gilt 2000 nM + AZD 200 nM
Gilt 2000 nM + AZD 300 nM
MRX 500 nM
MRX 1000 nM
MRX 2000 nM
MRX 1000 nM + AZD 200 nM
MRX 1000 nM + AZD 300 nM
MRX 2000 nM + AZD 200 nM
MRX 2000 nM + AZD 300 nM
THP-1 (FLT3-wt; 24 h)
Annexin V+/PI+
Annexin V+/PI-
CI<0.55
CI<0.72
Annexin V + cells (%)
Control
AZD 200 nM
AZD 300 nM
AZD 400 nM
Gilt 500 nM
Gilt 1000 nM
Gilt 2000 nM
Gilt 1000 nM + AZD 300 nM
Gilt 1000 nM + AZD 400 nM
Gilt 2000 nM + AZD 300 nM
Gilt 2000 nM + AZD 400 nM
MRX 500 nM
MRX 1000 nM
MRX 2000 nM
MRX 1000 nM + AZD 300 nM
MRX 1000 nM + AZD 400 nM
MRX 2000 nM + AZD 300 nM
MRX 2000 nM + AZD 400 nM

Figure S2

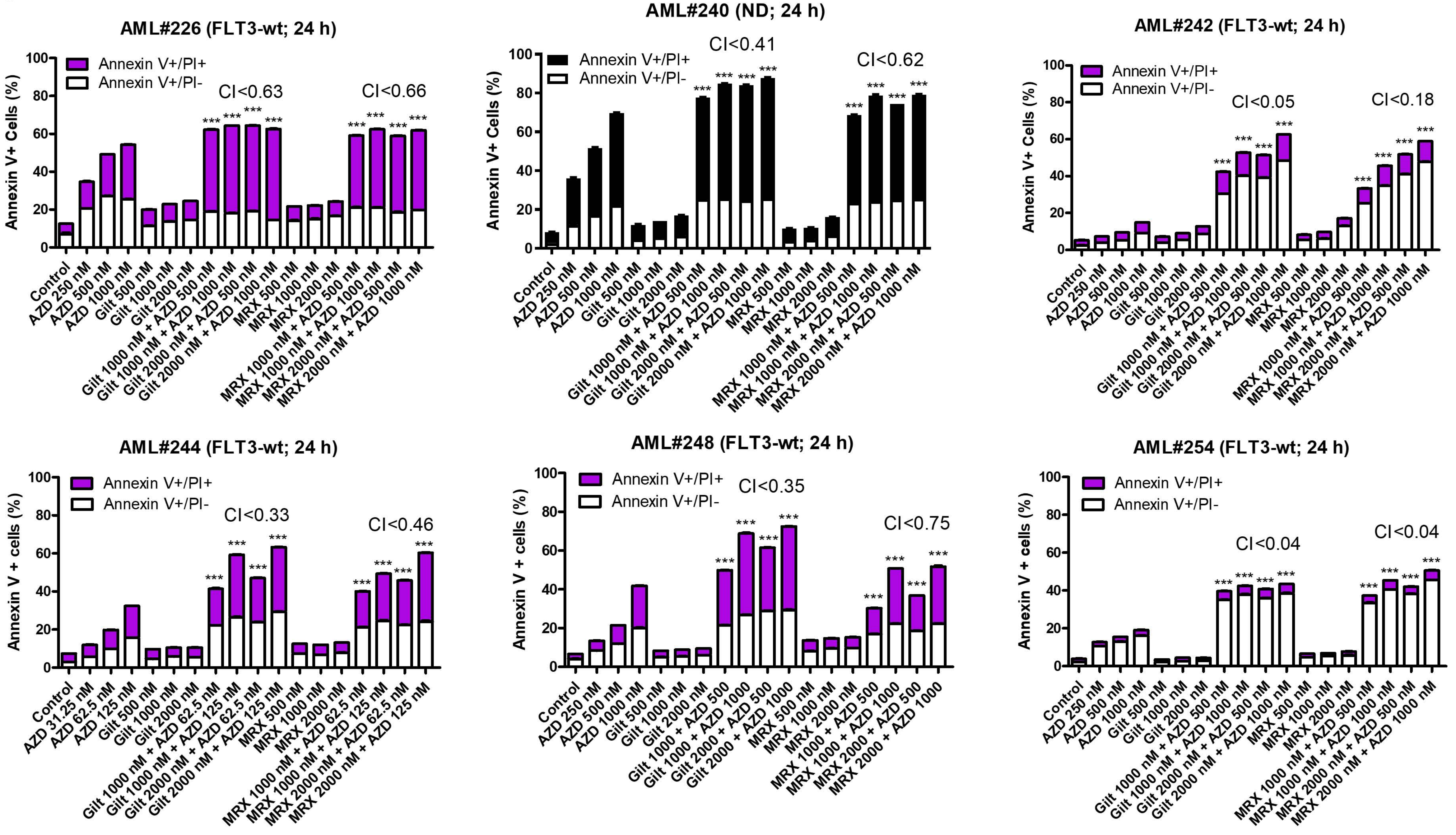

AML#226 (FLT3-wt; 24 h)
Annexin V+/PI+
Annexin V+/PI-
CI<0.63
CI<0.66
Annexin V+ Cells (%)
0
20
40
60
80
100
Control
AZD 250 nM
AZD 500 nM
AZD 1000 nM
Gilt 500 nM
Gilt 1000 nM
Gilt 2000 nM
Gilt 1000 nM + AZD 500 nM
Gilt 1000 nM + AZD 1000 nM
Gilt 2000 nM + AZD 500 nM
Gilt 2000 nM + AZD 1000 nM
MRX 500 nM
MRX 1000 nM
MRX 2000 nM
MRX 1000 nM + AZD 500 nM
MRX 1000 nM + AZD 1000 nM
MRX 2000 nM + AZD 500 nM
MRX 2000 nM + AZD 1000 nM
AML#240 (ND; 24 h)
Annexin V+/PI+
Annexin V+/PI-
CI<0.41
CI<0.62
Annexin V+ Cells (%)
AML#242 (FLT3-wt; 24 h)
Annexin V+/PI+
Annexin V+/PI-
CI<0.05
CI<0.18
Annexin V+ Cells (%)
AML#244 (FLT3-wt; 24 h)
Annexin V+/PI+
Annexin V+/PI-
CI<0.33
CI<0.46
Annexin V + cells (%)
Control
AZD 31.25 nM
AZD 62.5 nM
AZD 125 nM
Gilt 500 nM
Gilt 1000 nM
Gilt 2000 nM
Gilt 1000 nM + AZD 62.5 nM
Gilt 1000 nM + AZD 125 nM
Gilt 2000 nM + AZD 62.5 nM
Gilt 2000 nM + AZD 125 nM
MRX 500 nM
MRX 1000 nM
MRX 2000 nM
MRX 1000 nM + AZD 62.5 nM
MRX 1000 nM + AZD 125 nM
MRX 2000 nM + AZD 62.5 nM
MRX 2000 nM + AZD 125 nM
AML#248 (FLT3-wt; 24 h)
Annexin V+/PI+
Annexin V+/PI-
CI<0.35
CI<0.75
Annexin V + cells (%)
Control
AZD 250 nM
AZD 500 nM
AZD 1000 nM
Gilt 500 nM
Gilt 1000 nM
Gilt 2000 nM
Gilt 1000 + AZD 500
Gilt 1000 + AZD 1000
Gilt 2000 + AZD 500
Gilt 2000 + AZD 1000
MRX 500 nM
MRX 1000 nM
MRX 2000 nM
MRX 1000 + AZD 500
MRX 1000 + AZD 1000
MRX 2000 + AZD 500
MRX 2000 + AZD 1000
AML#254 (FLT3-wt; 24 h)
Annexin V+/PI+
Annexin V+/PI-
CI<0.04
CI<0.04
Annexin V + cells (%)
***

Figure S3

A

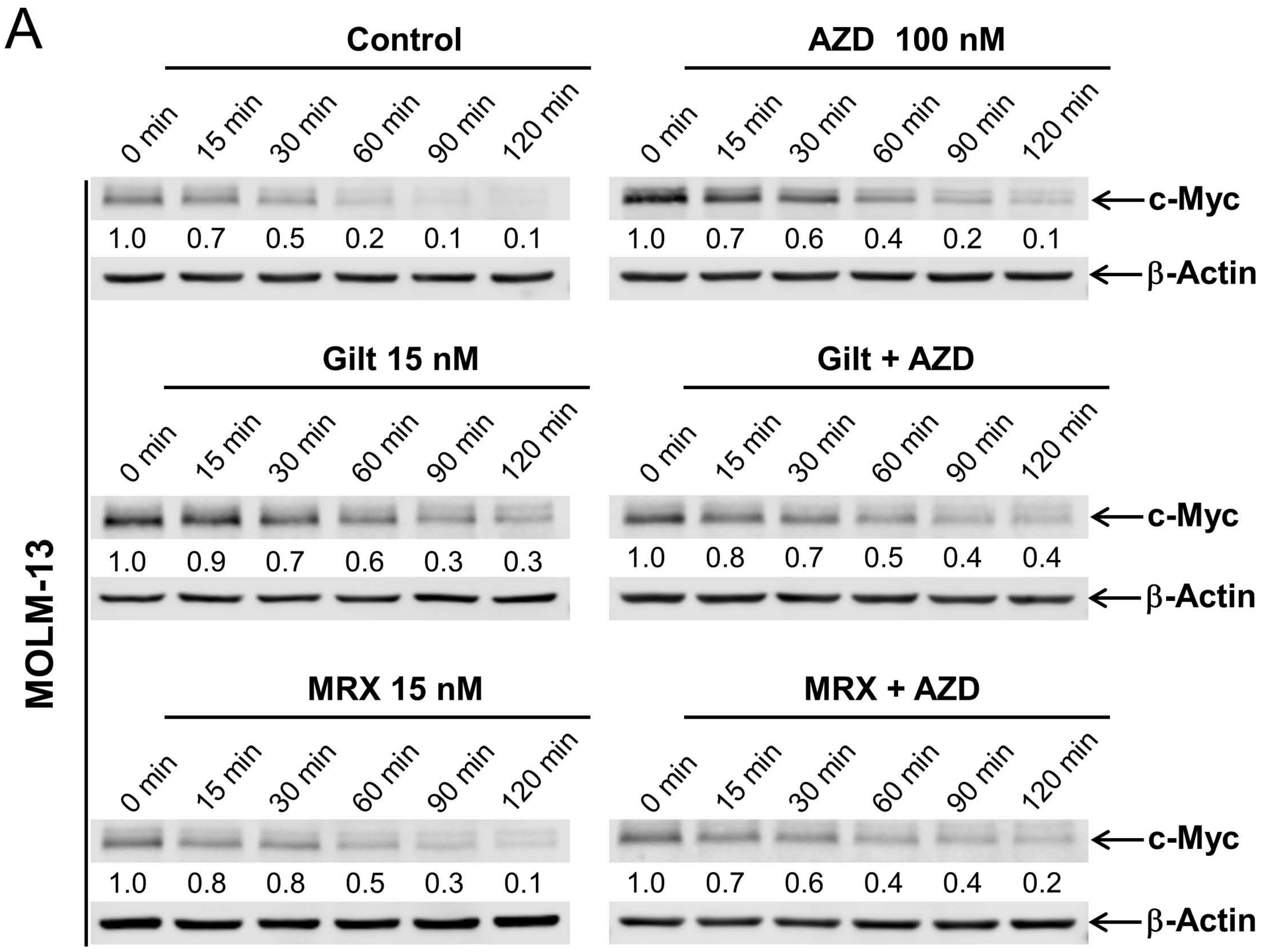

B

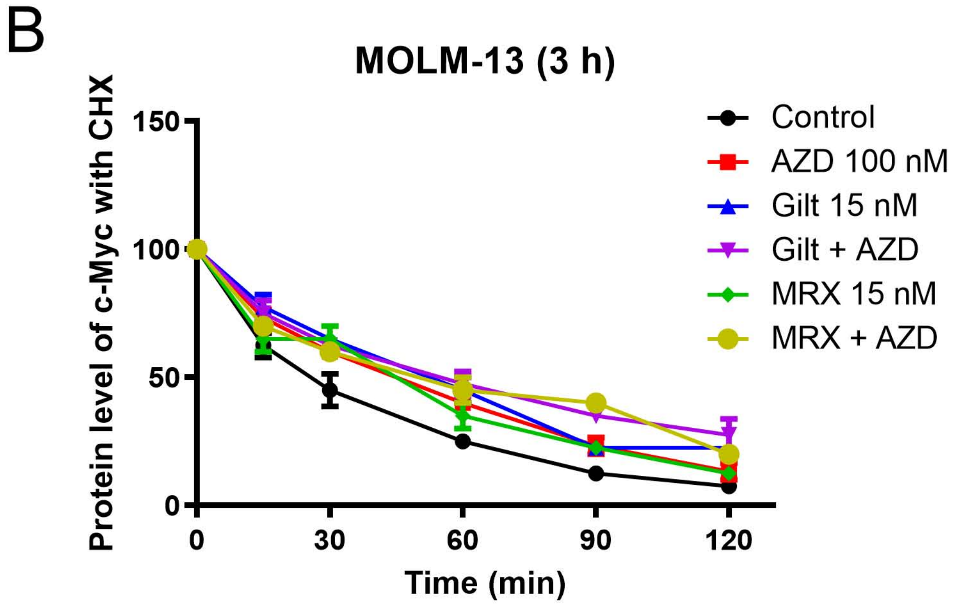

C

c-Myc protein half-life in MOLM-13 cells post drug treatment						
**Experimental condition**	Control	AZD 100 nM	Gilt 15 nM	Gilt + AZD	MRX 15 nM	MRX + AZD
**c-Myc $t_{1/2}$ (min)**	24	38	45	51	35	44

Figure S4A-C

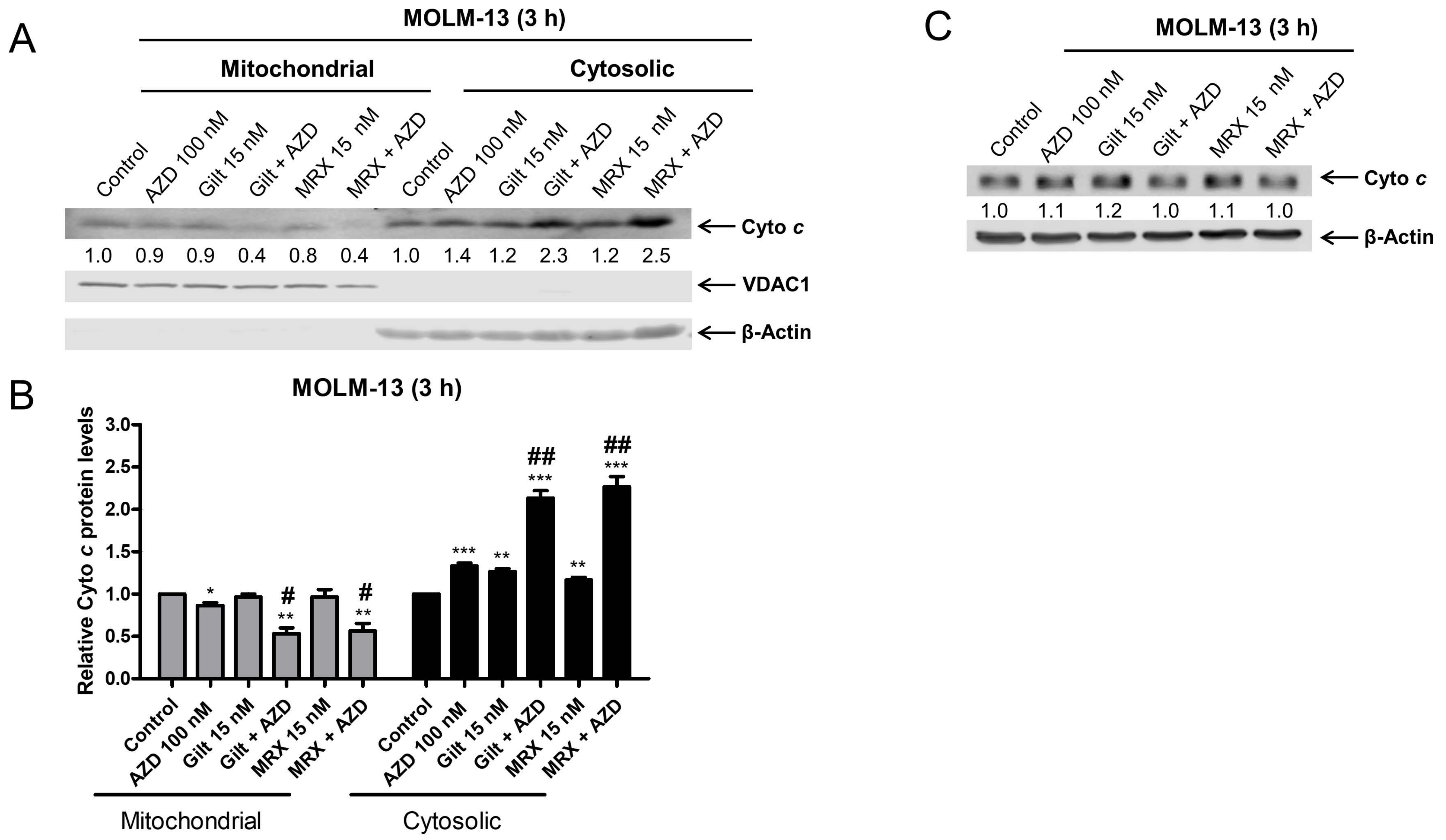
A
MOLM-13 (3 h)
Mitochondrial
Cytosolic
Control
AZD 100 nM
Gilt 15 nM
Gilt + AZD
MRX 15 nM
MRX + AZD
Cyto c
1.0 0.9 0.9 0.4 0.8 0.4 1.0 1.4 1.2 2.3 1.2 2.5
VDAC1
β-Actin
B
MOLM-13 (3 h)
Relative Cyto c protein levels
3.0
2.5
2.0
1.5
1.0
0.5
0.0
Mitochondrial
Cytosolic
C
MOLM-13 (3 h)
Cyto c
1.0 1.1 1.2 1.0 1.1 1.0
β-Actin

Figure S4D-I

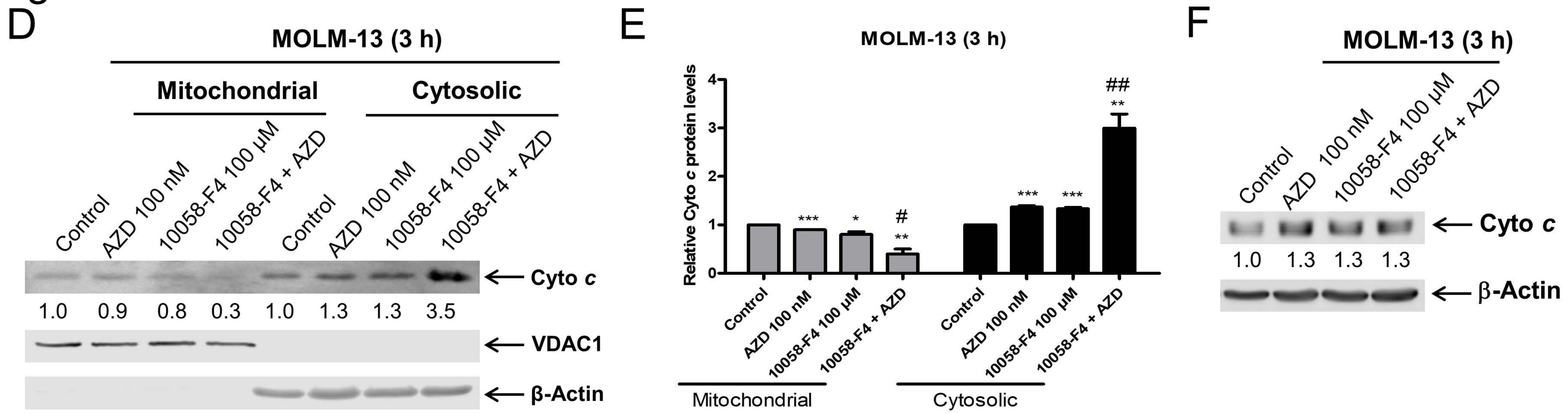
D
MOLM-13 (3 h)
Mitochondrial
Cytosolic
Control
AZD 100 nM
10058-F4 100 µM
10058-F4 + AZD
Control
AZD 100 nM
10058-F4 100 µM
10058-F4 + AZD
Cyto c
1.0 0.9 0.8 0.3 1.0 1.3 1.3 3.5
VDAC1
β-Actin
E
MOLM-13 (3 h)
Relative Cyto c protein levels
Control
AZD 100 nM
10058-F4 100 µM
10058-F4 + AZD
Mitochondrial
Cytosolic
F
MOLM-13 (3 h)
Control
AZD 100 nM
10058-F4 100 µM
10058-F4 + AZD
Cyto c
1.0 1.3 1.3 1.3
β-Actin

Figure S5

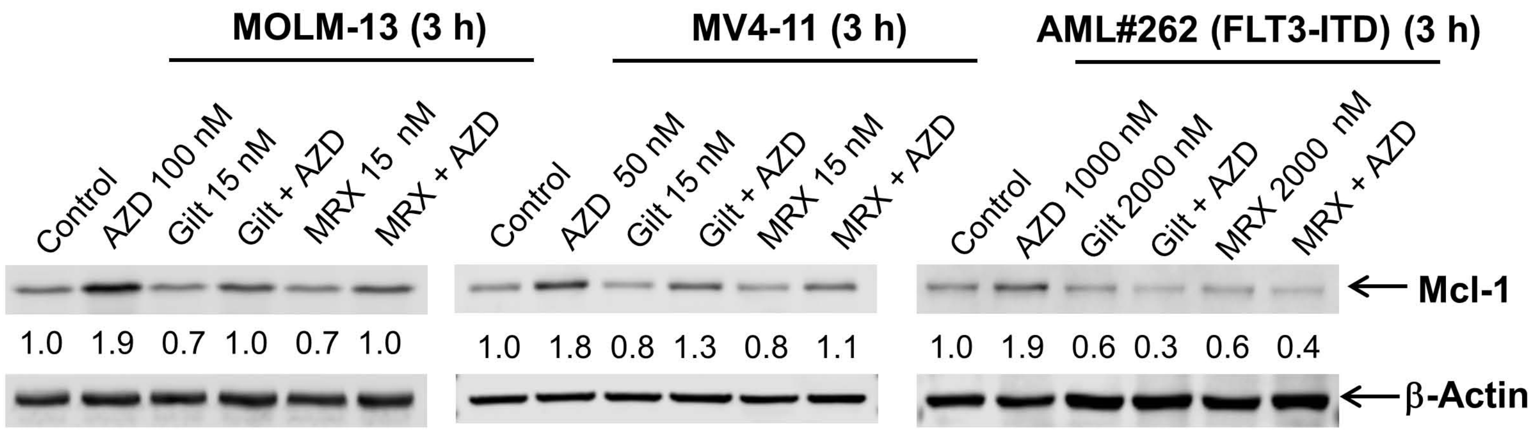

MOLM-13 (3 h)
MV4-11 (3 h)
AML#262 (FLT3-ITD) (3 h)
Control
AZD 100 nM
Gilt 15 nM
Gilt + AZD
MRX 15 nM
MRX + AZD
Control
AZD 50 nM
Gilt 15 nM
Gilt + AZD
MRX 15 nM
MRX + AZD
Control
AZD 1000 nM
Gilt 2000 nM
Gilt + AZD
MRX 2000 nM
MRX + AZD
Mcl-1
1.0 1.9 0.7 1.0 0.7 1.0
1.0 1.8 0.8 1.3 0.8 1.1
1.0 1.9 0.6 0.3 0.6 0.4
β-Actin

Figure S6

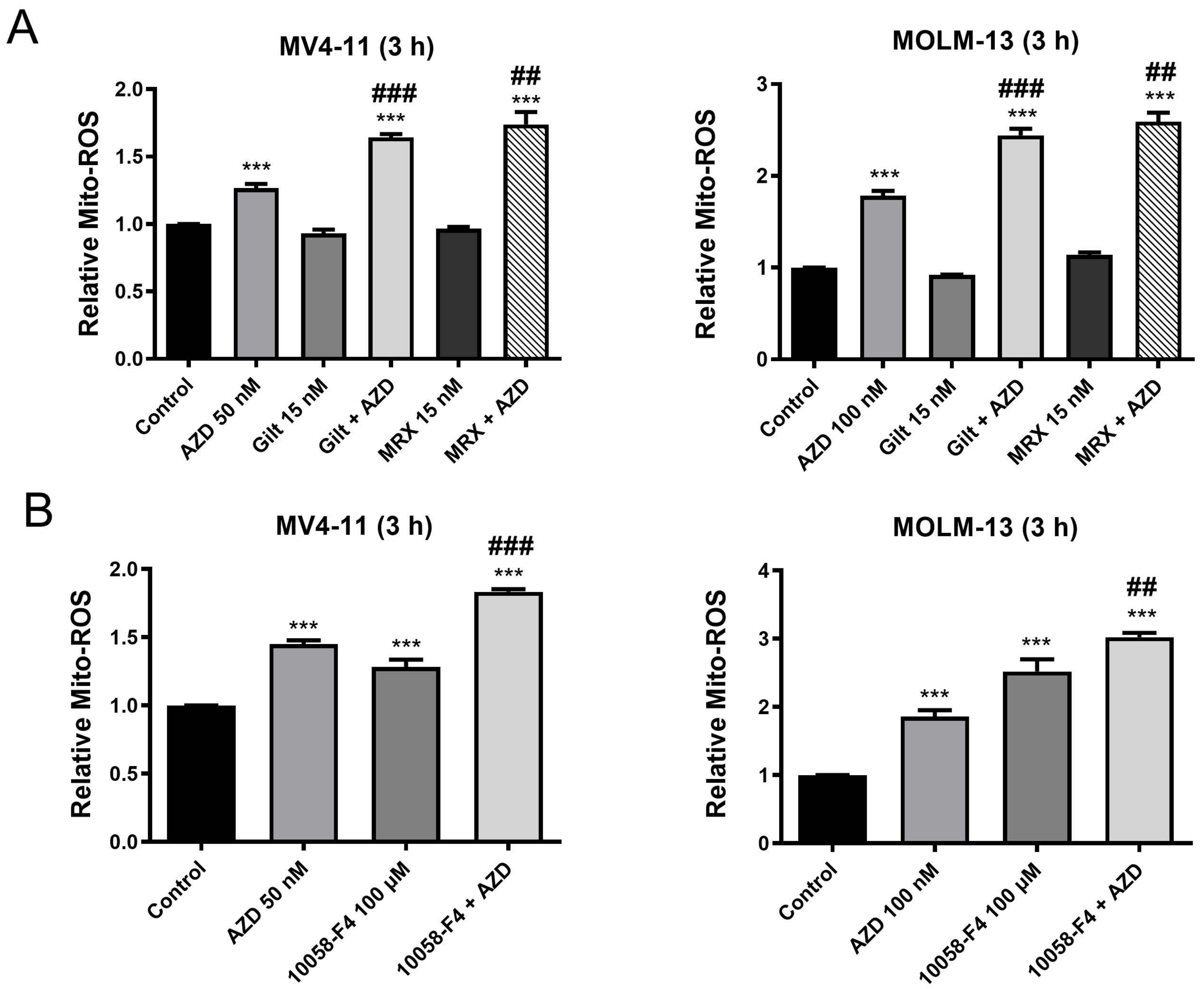
A
MV4-11 (3 h)
Relative Mito-ROS
2.0
1.5
1.0
0.5
0.0
***
###
***
##
***
Control
AZD 50 nM
Gilt 15 nM
Gilt + AZD
MRX 15 nM
MRX + AZD
MOLM-13 (3 h)
Relative Mito-ROS
3
2
1
0
***
###
***
##
***
Control
AZD 100 nM
Gilt 15 nM
Gilt + AZD
MRX 15 nM
MRX + AZD
B
MV4-11 (3 h)
Relative Mito-ROS
2.0
1.5
1.0
0.5
0.0
***
***
###
***
Control
AZD 50 nM
10058-F4 100 µM
10058-F4 + AZD
MOLM-13 (3 h)
Relative Mito-ROS
4
3
2
1
0
***
***
##
***
Control
AZD 100 nM
10058-F4 100 µM
10058-F4 + AZD

Table S1. Patient characteristics of the primary AML patient samples used in this study

Patient	Gender	Age (year)	Disease status	FAB subtype	Cytogenetics	Blast purity (%)	Gene mutation/Fusion gene
AML#213	Male	3	Newly diagnosed	M4/M5	46, XY	64	FLT3-ITD
AML#213	Male	3	Newly diagnosed	M4/M5	46, XY	64	FLT3-ITD
AML#217	Male	66	Newly diagnosed	M2	47, XY, +13/46, XY	94	FLT3-ITD, NPM1, TET2
AML#226	Female	64	Newly diagnosed	AML	46, XX	87	CEBPA
AML#240	Male	8	Newly diagnosed	AML	46, XY, inv(16)(p13q22)/47, XY, +8,inv(16)(p13q22)	78.5	NA
AML#242	Male	63	Newly diagnosed	M3	46, XY, t(15;17)(q24;q21)/46, XY	91	PML/RARa, WT1, PRAME
AML#244	Male	50	Newly diagnosed	AML	47, XY, t(4;11)(q21;q23), +6	90.5	negative
AML#245	Female	31	Newly diagnosed	M3	46, XX, t(15;17)(q24;q21)/46,XX	97	FLT3-ITD, PML/RARa, WT1, PRAME
AML#246	Male	38	Newly diagnosed	AML	46, XY	77.5	FLT3-ITD, FLT3-TKD, MLL-PTD
AML#248	Male	16	Newly diagnosed	AML	45, X,-Y	79	CEBPA, NRAS, PTPN11
AML#254	Female	15	Newly diagnosed	M5	46, XX	78	MLL-AF9, KMT2A, NF1
AML#256	Male	57	Newly diagnosed	M4/M5	47, XY, +8	80.5	FLT3-TKD, DNMT3A, SH2B3, TCF, RUNX1, MLL-PTD
AML#257	Female	53	Newly diagnosed	M4/M5	46, XX	94.5	FLT3-ITD, NPM1, TET2
AML#262	Male	57	Newly diagnosed	AML	46,XY	70.5%	FLT3-ITD, DNMT3A, IDH2, dupMLL
